# Activation of Proinflammatory Responses in Cells of the Airway Mucosa by Particulate Matter: Oxidant- and Non-Oxidant-Mediated Triggering Mechanisms

**DOI:** 10.3390/biom5031399

**Published:** 2015-07-02

**Authors:** Johan Øvrevik, Magne Refsnes, Marit Låg, Jørn A. Holme, Per E. Schwarze

**Affiliations:** Department of Air Pollution and Noise, Division of Environmental Medicine, Norwegian Institute of Public Health, P.O. Box 4404 Nydalen, N-0403 Oslo, Norway; E-Mails: magne.refsnes@fhi.no (M.R.); marit.lag@fhi.no (M.L.); jorn.holme@fhi.no (J.A.H.); per.schwarze@fhi.no (P.E.S.)

**Keywords:** particles, fibers, particulate matter, nanoparticles, silica, asbestos, quartz, receptors, inflammasome, ROS, oxidants, transcription factors, cytokines

## Abstract

Inflammation is considered to play a central role in a diverse range of disease outcomes associated with exposure to various types of inhalable particulates. The initial mechanisms through which particles trigger cellular responses leading to activation of inflammatory responses are crucial to clarify in order to understand what physico-chemical characteristics govern the inflammogenic activity of particulate matter and why some particles are more harmful than others. Recent research suggests that molecular triggering mechanisms involved in activation of proinflammatory genes and onset of inflammatory reactions by particles or soluble particle components can be categorized into direct formation of reactive oxygen species (ROS) with subsequent oxidative stress, interaction with the lipid layer of cellular membranes, activation of cell surface receptors, and direct interactions with intracellular molecular targets. The present review focuses on the immediate effects and responses in cells exposed to particles and central down-stream signaling mechanisms involved in regulation of proinflammatory genes, with special emphasis on the role of oxidant and non-oxidant triggering mechanisms. Importantly, ROS act as a central second-messenger in a variety of signaling pathways. Even non-oxidant mediated triggering mechanisms are therefore also likely to activate downstream redox-regulated events.

## 1. Introduction

Particle toxicology covers effects of a broad range of different compounds, including “classical” mineral particles and fibers, complex stone particles and windblown soil dusts, to airborne particulate matter (PM) in polluted cities, and engineered nanomaterials [1,2]. Inhalation of airborne particles and fibers of various origins has been associated with the development or exacerbation of a number of pathological conditions including asthma, chronic obstructive pulmonary disease (COPD), pulmonary fibrosis (including silicosis and asbestosis), cancer, cardiovascular disease, and increased susceptibility towards respiratory infections [1,3,4,5,6,7,8]. Recent studies on ambient air PM also suggest possible links with development of obesity, type-2 diabetes, and neurodegenerative diseases [9,10,11]. As pointed out by Donaldson *et al.* [12], different classes of particles clearly induce distinctly different pathologies. Thus, particle-induced diseases clearly cannot be attributed to a single causing factor, but rather arise from a multitude of different mechanisms. Nevertheless, the diverse range of adverse health effects associated with inhalation of airborne particulates shares the involvement of a common pathological condition: inflammation.

Inflammation is considered a central mechanism for development of health effects by particle exposure [4,13,14]. There is compelling evidence of a strong causal relationship between induction or exacerbation of inflammatory responses in the airway mucosa, and induction or exacerbation of respiratory disease by PM exposure [4,6,13,15,16,17]. Moreover, inflammatory responses are considered central in development of fibrosis and cancer from mineral particles and fibers such as quartz and asbestos [3,16,18]. Pulmonary inflammation is also proposed to be a possible causal factor involved in the cardiovascular effects from PM exposure. Inflammatory responses in the airways may result in the release of cytokines and other proinflammatory or pro-thrombotic mediators into the circulation, leading to arterial remodeling or affecting plaque stability in arterial walls [14,19,20,21]. Thus, understanding how particles trigger inflammatory reactions in the airways is a central issue in particle toxicology.

A number of highly varying endogenous and environmental stimuli, including particulates, may activate intracellular signaling cascades in the cells of the airways, triggering transcriptional activation of proinflammatory genes. Early signaling events typically involve activation of various receptor tyrosine kinases (RTKs), G-protein coupled receptors (GPCRs), and/or oxidative stress. Non-receptor tyrosine kinases such as Src and Syk, Rac GTPases, and Ras family proteins subsequently turn on down-stream signaling pathways. The nuclear factor-κB (NF-κB) represents the quintessential transcriptional regulator of proinflammatory responses. The classical NF-κB-pathway typically consists of the p65/p50 dimer which binds to κB-sites in the promoter region of a variety of proinflammatory genes including several cytokines and chemokines [7,8]. In unstimulated cells, NF-κB is kept inactive in the cytosol by the inhibitor of κB (IκB) and activated by upstream IκB kinases (IKKs). Other central transcription factors involved in regulation of proinflammatory genes include activator protein-1 (AP-1), CCAAT-enhancer-binding protein (C/EBP), interferon regulatory factors (IRFs), and the signal transducer and regulator of transduction (STAT), which is part of the JAK-STAT pathway. The mitogen-activated protein kinase (MAPK) family of serine/threonine kinases represents another group of signaling mediators that are almost ubiquitously involved in regulation of inflammatory responses. The best described MAPK members are the extracellular signal-regulated kinase-1 and -2 (ERK1/2), the c-Jun-N-terminal kinases (JNKs), and the p38 MAPKs (Puddicombe and Davies, 2000). MAPKs are activated in response to a range of extracellular stimuli (growth factors, cytokines, hormones, oxidants, toxins, physical stress) and regulate a variety of cellular responses including immune activation and inflammation. The ERK1/2 and JNK cascades typically activate transcription factors such as activator protein-1 (AP-1), while p38 has often been implicated in mRNA stabilization [22]. Along with a variety of other signaling mechanisms, including calcium signaling and cyclic AMP (cAMP), these pathways regulate and coordinate the expression and release of a variety of mediators, such as cytokines, chemokines, and adhesion factors, which orchestrate the resolution of the inflammatory response [13,23].

In the lung, particles may interact with the lung lining fluid and the cells of the airways. Pulmonary epithelial cells and resident macrophages are considered the primary targets of inhaled pollutants such as PM, but deposited particles also affect sensory neurons, dendritic cells, and other immune cells. Their initial responses upon particle exposure are crucial in the onset and regulation of both innate and adaptive immune responses. These effects may derive from interactions with the cellular plasma membrane and its receptors and ion channels, or with intracellular targets, that directly or indirectly trigger responses leading to transcriptional activation of proinflammatory genes. Identifying these initiating molecular events and how they are linked to regulation of proinflammatory genes is central to understand how particles trigger their harmful effects, and to identify the particle determinants that are crucial for inflammatory responses.

The main focus of this review is the initiating molecular triggering mechanisms and the down-stream intermediate signaling events that ultimately lead to transcriptional activation of proinflammatory genes when cells of the airway mucosa are exposed to particles. The role of reactive oxygen species (ROS) and oxidative stress is discussed in detail, as this has represented a central paradigm for the toxicity and proinflammatory effects of particle exposure. To restrict an already comprehensive topic, this review will predominately deal with particles originating intentionally or unintentionally from human activities, while pure biological particles such as fungal spores and pollen are not included. However, the role of bacterial endotoxins in ambient PM will be discussed to some extent.

## 2. Some General Principles of Particle Toxicology

Airborne, inhalable particulates constitute a heterogeneous, complex group of compounds from various sources. A number of factors affect the toxicity of particles, including size, shape, structure, surface reactivity, solubility/biopersistence, and presence of soluble components. Small particles are generally more toxic than larger particles due to a larger surface-to-mass ratio. Size also affects deposition in the airways. Although there is no straightforward correlation between particle size and deposition patterns, smaller particles tend to penetrate deeper and deposit more efficiently in the airways. With respect to air pollution the most important size-fractions are PM_10_ (<10 µm; respirable particles), PM_10–2.5_ (10–2.5 µm; coarse particles), PM_2.5_ (<2.5 µm; fine particles) and PM_0.1_ (<0.1 µm; ultrafine particles). Coarse particles in urban air generally originate from wear-processes, such as road and tire abrasion, construction work, or from natural windblown dusts. The fine and ultrafine fractions tend to be dominated by particles from combustion sources. Shape and physicochemical structure also affect the toxicity of particles. Long, thin fibers (>15 µm) are more hazardous than short fibers or spherical particles, because they cannot be completely engulfed and removed by alveolar macrophages [16]. Crystalline particles such as α-quartz (SiO_2_) are generally more toxic than amorphous particles of identical chemical composition and may induce a stronger up-regulation of proinflammatory genes [24,25]. Surface reactivity, such as surface charge or presence of reactive groups (such as silanol groups on quartz) or redox-active transition metals may also affect the toxicity of particles. Amongst others this may contribute to the formation of ROS which is considered a central mechanism for particle toxicity. Similarly, the presence of soluble toxic components on the particle surface, such as metal ions, organic compounds including polycyclic aromatic hydrocarbons (PAHs) and biological materials such as bacterial endotoxins or allergens are also important mediators of adverse effects from particle exposure. Retention and biopersistence are also important factors, as particles retained for longer periods in the lung (or other organs) may promote more damage. For the same reason, soluble particles tend to be less harmful than insoluble particles unless they contain particularly toxic chemicals. From a toxicological point of view, particles can be divided in four broad categories based on their physicochemical characteristics and mechanisms of effects: (1) Solid (insoluble) particles, which mainly mediate effects through surface reactivity. These include various mineral particles such as crystalline silica (quartz) and solid nanoparticles. (2) Fibers, for which fiber length, diameter and biopersistence are the three main parameters of concern. These include classical mineral fibers such as asbestos, but also the more novel nanofibers such as single- and multi-walled carbon nanotubes. (3) Soluble particles, typically metal oxides such as ZnO and CuO, which mediate effects by release of reactive ions. (4) Complex particles, which contain various active toxic compounds. This last group includes ambient PM as well as combustion particles from specific sources, such as diesel exhaust and wood smoke particles (DEP and WSP). In contrast to the solid particles and fibers as well as soluble metal oxides, the triggering mechanisms of combustion-derived particles and ambient PM are likely to be considerably more complex. DEP alone, which may constitute a considerable fraction of fine and ultrafine PM, carry an abundance of different organic chemicals, as well as embedded metallic ashes [26,27]. Several studies show clearly that the majority of effects from DEP are due to its soluble constituents [27,28,29,30,31,32,33,34]. However, additional effects from the carbon core should not be excluded. It is generally accepted that the effects of DEP and other combustion particles are due to both the soluble fraction and the insoluble carbon core, amongst others because the particles act as a carrier of other toxic substances [35]. Thus, particle-induced effects may be attributed to responses triggered by particle surface components, by soluble constituents leaking from the particles, or by ROS generated by the particle or particle components.

## 3. Molecular Initiating Events: Triggering Mechanisms for Particle-Induced Activation of Proinflammatory Genes

### 3.1. Oxidative Stress and Direct ROS Formation by Particles

**Figure 1:**
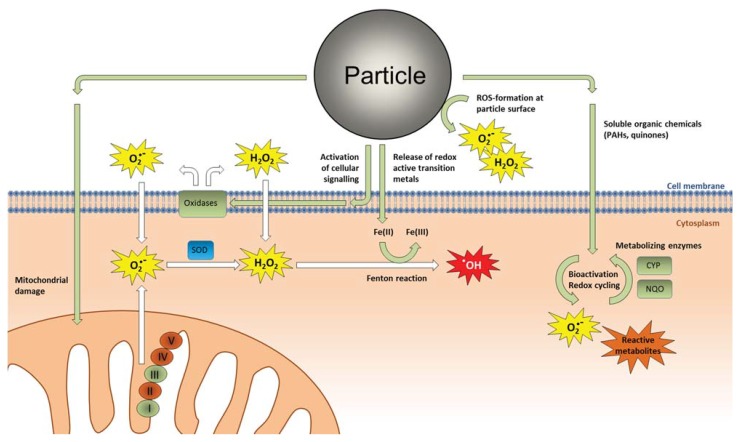
Potential sources of reactive oxygen species (ROS) formation in particle-exposed cells. Interpreting the effects of antioxidants on cellular responses from particle exposure is inherently difficult due to the many potential sources of ROS. ROS may be generated directly by reactive particle surfaces in contact with aqueous media. Soluble organic constituents such as PAHs, and quinones may form ROS and reactive electrophilic metabolites through redox cycling and metabolic activation. Fenton-reactive transition metals may contribute to the formation of highly reactive hydroxyl radicals (^●^OH). Activation of intracellular signaling pathways may trigger production of superoxide (O_2_^●−^) and hydrogen peroxide (H_2_O_2_) through activation of membrane bound oxidases, and damage to mitochondria may lead to superoxide production. Abbreviations: CYP, cytochrome P450; NQO, NAD(P)H:quinone oxidoreductase; SOD, superoxide dismutase.

A dominating theory has been that different types of particles trigger proinflammatory responses and other cellular effects through formation of ROS and induction of oxidative stress. The roles of ROS formation and oxidative stress in particle toxicology have been extensively reviewed in previous papers [17,36,37,38,39,40,41,42,43,44,45,46]. In its strictest form, the ROS hypothesis suggests that the ability of particles to induce ROS formation in abiotic/cell-free environments, such as water, buffers, or cell culture media can predict a particle’s relative potential to induce toxicity in cells, animals and humans [44,45,47,48]. The oxidative capacity or the ability to induce formation of ROS in cell-free systems has therefore been suggested as a possible screening method to assess the biological reactivity of particles [47,48]. In a broader context, the “oxidative stress paradigm” in particle toxicology encompasses both primary ROS generation by the particles and particle components as well as secondary formation of ROS and reactive nitrogen species (RNS) by particle-exposed cells (Figure 1). The latter represents an effect of exposure and is therefore not a true triggering mechanism for effects, as pointed out in earlier reviews [49,50]. According to the oxidative stress paradigm, the cells responds to low levels of oxidative stress by increasing antioxidant levels and restoring cellular redox homeostasis amongst others through activation of the ROS-sensitive Nrf2/ARE pathway [51,52]. At intermediate levels, oxidative stress leads to activation of MAPKs and central proinflammatory transcription factors such as NF-κB and AP-1 leading to up-regulation of proinflammatory genes including various cytokines and chemokines. At high levels, oxidative stress results in toxicity amongst others through perturbation of the mitochondrial permeability transition pore and disruption of the electron transport chain resulting in cellular apoptosis or necrosis [17,35]. In line with this concept, several studies have shown that anti-oxidants attenuate various particle-induced responses including activation of protein kinases and transcription factors involved in regulation of proinflammatory genes [53,54,55,56,57,58,59]. This line of evidence suggests that ROS is of major importance to many of the observed cellular responses from particle exposure. Nevertheless, it is often difficult to determine with certainty whether such effects of antioxidant treatments are due to interference with primary particle-derived ROS formation or secondary endogenous ROS generation by the cells. A range of studies has also failed to find a consistent correlation between oxidative capacity of particles in cell-free systems and their ability to induce inflammatory responses or other effects in cells, animals or humans [60,61,62,63,64,65,66,67,68,69,70,71,72,73,74,75,76,77,78,79,80,81,82]. This probably reflects that transcriptional activation of proinflammatory genes by particle exposure rather arises from non-oxidant triggering mechanisms, although one cannot exclude that the assays used for oxidative capacity assessment were inadequate. Combustion particles and other particulates carrying organic chemicals may clearly induce ROS formation through redox-cycling of quinone species and metabolic degradation of for instance PAHs, which has been firmly linked to oxidative damage on macromolecules [17,41]. Similarly, the presence of transition metals such as iron and copper might undergo redox cycling (Fenton or Haber-Weiss reactions) and contribute to ROS generation. However, the relative contribution of this more direct ROS formation from PM-bound chemicals and metals compared to endogenous ROS generation by activated cell cellular enzymes (as discussed later in this review) remains unclear, especially when it comes to inflammatory effects from relevant real-life (low-level) exposure-concentrations of airborne particulates. In any case, solid evidence supporting a role of primary/direct particle-derived ROS formation as a main triggering mechanism for particle-induced inflammation is lacking, and inhalable particulates clearly also trigger effects through other non-oxidant-mediated mechanisms. An overview of the potential sources of ROS-formation and oxidative stress in particle-exposed cells is given in Figure 1.

### 3.2. Interactions with Cellular Membranes

More than 50 years ago it became evident that quartz and asbestos possessed lytic action on cellular membranes [83,84,85,86]. The pulmonary toxicity of quartz was proposed to be due to its continuous killing of macrophage by lysosomal rupture and release of lysosomal enzymes [84,87,88]. Inhaled quartz particles deposited in the airways are rapidly coated by lung surfactant, masking their reactive sites and reducing their toxicity. However, upon phagocytosis by macrophages, particles enter the acidic environment of the phagolysosomes where the protective coating is stripped off. The quartz particles are then free to interact with the lysosomal membranes, ultimately leading to lysosomal rupture, cell death and release of particles along with the reactive lysosomal content into the lung environment [89]. In extension of these observations of the effects of quartz on macrophages, the molecular basis of gouty inflammation was suggested to be due to interactions between monosodium urate (MSU) crystals and secondary lysosomes formed in response to phagocytosis of MSU crystals by lymphocytes [90]. This intriguing similarity between quartz toxicity and gout pathogenesis has recently revitalized the mechanistic understanding of how solid particulates trigger inflammation in macrophages and other immune cells, as will be discussed later. The ability to induce hemolysis of red blood cells (RBC) has long been considered as a relevant screen to assess mineral particle toxicity that could mimic the effects seen in macrophage lysosomes [91]. More recent findings suggest that the RBC-hemolysis assay may provide good prediction of *in vivo* inflammatory effects from mineral particles and solid nanoparticles [63,69,92,93,94]. These findings further suggest that membrane rupture may be an initiating triggering mechanism for particle-induced effects, ultimately leading to transcriptional activation of proinflammatory genes.

**Figure 2:**
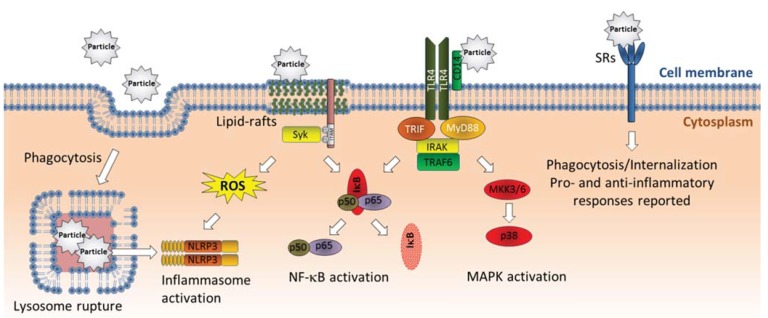
Non-oxidant triggering mechanisms for activation of proinflammatory genes by solid particles. Various solid particles have been found to interact directly with cellular membranes. Phagocytosed particles may damage lysosomal membranes, leading to lysosomal leakage and activation of the NLRP3 inflammasome with subsequent cleavage/release of IL-1β. Crystalline particles may also interact directly with cholesterol in lipid rafts, leading to lipid sorting and Syk-activation, possibly through nonspecific aggregation and activation of receptors in rafts. Syk activation may possibly activate IL-1β and other proinflammatory genes through ROS-mediated NLRP3 activation as well as stimulation of NF-κB signaling. Crystalline particles have been reported to bind CD14, which may stimulate cytokine responses through activation of TLR4-mediated activation of NF-κB and MAPK signaling. Finally, several solid particles have been reported to be bound by scavenger receptors which appear to result in both pro- and anti-inflammatory effects depending on cell type. Abbreviations: IκB, inhibitor of κB; IRAK, interleukin-1 receptor-associated kinase; MKK3/6, mitogen activated protein kinase kinase-3/-6; MyD88, myeloid differentiation primary response gene 88; NF-κB, nuclear factor-κB; NRLP3, NACHT, LRR and PYD domains-containing protein 3; ROS, reactive oxygen species; SRs, scavenger receptors; TLR4, Toll-like receptor 4; TRAF6, TNF receptor associated factor 6; TIR-domain-containing adapter-inducing interferon-β.

Effects of crystalline silica and other solid particles on intracellular signaling pathways regulating proinflammatory genes are clearly not restricted to phagocytic cells and may occur independently of membrane rupture. Crystalline silica appear to induce phosphorylation of MAPKs and Src-family tyrosin kinases involved in cytokine/chemokine responses in airway epithelial cells (A549) prior to particle uptake [95]. This suggests that these signaling events could be triggered through interactions between silica particles and membrane components. Of particular interest, Shi and co-workers reported that MSU crystals induced lipid sorting, clustering of cholesterol-rich lipid rafts, activation of Syk kinase and release of IL-1β in dendritic cells through receptor-independent membrane binding [96]. By attaching MSU crystals to the cantilever of an atomic-force microscope, they measured the strength of association between MSU crystals and the membranes of individual cells. The authors found that MSU crystals formed tight intermolecular bonding with cholesterol in the cell membrane and suggested that this may have led to nonspecific aggregation and activation of cellular receptors in rafts which subsequently may have activated Syk and stimulated release of IL-1β [96]. Syk-activation has more recently been shown to induce both IL-1β-transcription and cleavage of pro-IL-1β to mature IL-1β through combined activation of NF-κB and ROS-mediated stimulation of the NLRP3/caspase-1 [97]. Shi and colleagues later reported that the adjuvance of alum (crystal structures of aluminum containing salts) could also be attributed to similar direct interactions with membrane lipids in dendritic cells [98]. Moreover, carbon nanoparticles have been reported to alter the composition of lipid rafts in airway epithelial cells, by increasing the content of ceramides (sphingolipids) [99]. The increased ceramide levels resulted in activation of Src-family kinases and the EGF-receptor. The relevance of these events in *in vivo* inflammation was further confirmed by intervention therapies [99]. Although, the study did not confirm that carbon nanoparticles triggered these effects through direct interactions with membrane components, it seems likely that different solid particles and nanoparticles may induce effects at least partly through interactions with the lipid layer of cellular membranes (Figure 2).

Nevertheless, much still remain to be clarified regarding the exact molecular mechanisms through which crystalline particles damage the lysosomal membrane and other cellular membranes. Early studies indicated that hydrogen bonding between the silic acid on the surface of silica particles and phospholipids in the cell membrane increase membrane fluidity and permeability, ultimately leading to membrane rupture [91]. ROS formation by the particle surface has also been suggested as a potential mechanism for silica-induced membrane rupture [100]. However, lack of correlation between the ability to produce ROS and RBC-hemolysis indicates that membrane damage may primarily be triggered through other mechanisms [63,69,93]. More recently, it has been proposed that negatively charged groups on the surface of quartz and other oxides, interact with positively charged moieties in the head group of the phospholipids, consequently altering and disrupting the phospholipid bi-layer [89]. Significant membrane rupture may occur when lots of phospholipid head groups adsorb to the particle surface. The crystal structure itself has been suggested to be important in the process, possibly explaining differences in potency between different crystalline silica polymorphs [89]. However, negative surface charge may not be a prerequisite to damage biological membranes. As reviewed by Leroueil *et al.* [101], polycationic nanoparticles interact strongly with the lipid layer of cellular membranes and induce substantial membrane permeability. Moreover, in addition to charge, both particle size and shape may possibly also affect lysosomal destabilization [102,103].

**Figure 3:**
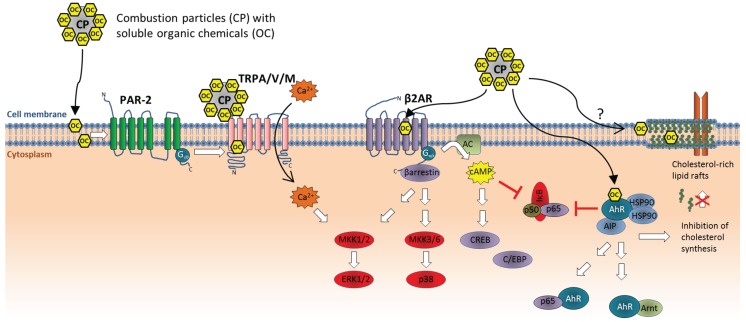
Non-oxidant triggering mechanisms for activation of proinflammatory genes by combustion particles. Combustion particles may activate TRPV1, TRPV4, and TRPM8 channels directly by their negative surface charge or through binding of soluble organic constituents. TRPA1 may be activated indirectly through activation of PAR-2 by soluble organics. TRP-channel activation mediates Ca^2+^-influx, which may activate proinflammatory genes, in part, through activation of ERK1/2. Cigarette smoke extracts and PAHs found in diesel exhaust and other combustion particles, may activate β2AR and stimulate cytokine responses, possibly through β-arrestin-mediated activation of MAPKs ERK1/2 and p38 or cAMP-induced activation of C/EBP and CRE-sites. However, cAMP appears to suppress NF-κB activity. PAHs and other dioxin-like constituents from combustion particles activate the AhR. AhR-activation may both suppress and induce proinflammatory responses depending on cell type and additional co-stimuli. AhR activation may inhibit NF-κB activation, but also bind to the p65 (RelA) subunit and activate κB-sites in the promotor region of proinflammatory genes. Certain cytokines genes have been reported to be regulated by the AhR:Arnt dimer, through XRE-sites. In addition, AhR:Arnt regulates CYP1-expression which may contribute to ROS formation through metabolic degradation of PAHs and other organic chemicals. AhR may also suppress cholesterol synthesis and affect membrane rafts, which may affect cellular signaling. Lipophilic constituents of combustion particles can also increase membrane fluidity and possibly affect rafts more directly. However, the role of lipid rafts and membrane fluidity in the proinflammatory effects from combustion particles remains to be determined. Abbreviations: AC, adenylate cyclase; AIP, AhR interacting protein; AhR, aryl hydrocarbon receptor; Arnt, AhR nuclear translocator; β2AR, β2-adrenergic receptor; Ca^2+^, calcium; cAMP, cyclic adenosine monophosphate; C/EBP, CCAAT-enhancer-binding proteins; CP, combustion particle; CREB, cAMP response element-binding protein; ERK1/2, extracellular signal regulated kinase-1/-2; HSP90, heat-shock protein 90; IκB, inhibitor of κB; MKK, mitogen activated protein kinase kinase; NF-κB, nuclear factor-κB; OC, organic chemicals; PAR-2, protease activated receptor-2; TRPA/V/M, transient receptor potential (TRP) cation channels A, V and M.

Compared to solid particles, such as mineral particles, fibers and nanoparticles, the importance of direct membrane interactions with ambient PM remains largely unexplored. Coarse PM (PM_10–2.5_), a crucial component in ambient PM, may contain a considerable amount of mineral particles, and some of these may potentially interact with cellular membranes in manners similar to those previously described for crystalline particles, nanoparticles, and endogenous crystals. Moreover, many of the cellular effects from combustion particles such as DEP (important in ambient PM) including activation of proinflammatory genes, appear mainly to be due to soluble organic constituents [27,28,29,30,31,32,33,34]. Studies by Lagadic-Gossmann and co-workers have shown that PAHs, which are found on most combustion particles, may alter the fluidity of cellular membranes and affect lipid-raft formation [104,105]. Since lipid rafts play a central role in aggregation of functional receptor-complexes this could affect a variety of signaling pathways and cellular functions. Notably, this response seems to be mediated through activation of the cytosolic aryl hydrocarbon receptor (AhR) and inhibition of cholesterol synthesis [104,105]. However, PAHs have also been found to increase the fluidity of cellular model membranes directly, and benzo[*a*]pyrene (B[*a*]P) may interact with carbonyl groups of phospholipids [106,107]. Increased membrane fluidity induced by ethanol exposure has been associated with suppression of innate immune responses and TLR4-signaling by affecting lipid rafts and lipid-protein interactions. However, chronic ethanol exposure may paradoxically enhance inflammatory processes which in part may result from adaptational processes rendering the cells more susceptible to other inflammogenic agents [108]. Thus, the possibility that soluble lipophilic constituents of DEP and other combustion-derived particles may affect regulation of proinflammatory genes at least partly through alteration of cell membrane fluidity (Figure 3) is an intriguing possibility that should be investigated.

### 3.3. Activation of Cell Surface Receptors

Membrane receptors represent another group of likely targets for particles and particle components. Indeed, several cell surface receptors have been implicated in particle-induced effects. It has long been known that crystalline silica and other particulates are bound by different scavenger receptors (SRs) [109,110,111,112]. SRs belong to a family of pattern-recognizing receptors that bind a broad range of polyanionic ligands with a certain spatial distribution of negative charges, and have thus been described as “molecular fly-papers” [113,114]. Their role in particle toxicity has mostly been linked to phagocytosis and cell death in macrophages. SRs might therefore play a role in particle internalization and processes preceding lysosomal rupture and subsequent inflammasome activation and IL-1β processing (as discussed under Chapter 4.2). However, a recent study shows that silica particles induce increased inflammasome activation and IL-1β release in alveolar macrophages lacking MARCO, the predominant silica-binding SR [115]. Moreover, the ability of silica to modulate TLR ligand-dependent activation of dendritic cells did not appear to depend on the class A SRs (SR-A) [116]. In contrast, knock-down of both MARCO and SR-A partially reduced silica-induced TNF-α responses and intracellular ROS generation in murine mast cells [117]. Thus, the role of SRs in particle toxicity may be highly cell-dependent, and their exact involvement in regulation of proinflammatory responses remains to be clarified (Figure 2). Other studies have suggested that asbestos fibers and air pollution particles may activate the EGF-receptor (EGFR) on the surface of epithelial cells leading to activation of ERK signaling and induction of cytokine responses [118,119,120]. However, EGFR-activation appears to be an indirect, down-stream effect of particle exposure, after cleavage of membrane bound EGFR-ligands in particle-exposed cells [95,121,122,123,124,125]. Another potential candidate is CD14 which has been reported to bind MSU crystals (Figure 2). CD14 is a co-receptor for many Toll-like receptors (TLRs), including TLR2 and TLR4, and known for its role in binding and presenting lipopolysaccharide (LPS) to TLR4. CD14 knock-down also suppressed MSU-induced p38 and NF-κB activation as well as release of IL-1β and CXCL1 in macrophages. These effects were mediated through TLR2/TLR4 signaling, and could possibly be of importance to other crystalline particles such as silica/quartz [126].

Veronesi and colleagues found that residual oil fly ash (ROFA) particles induced cytokine responses (IL-l-6, IL-8/CXCL8 and TNF-α) in bronchial epithelial cells (BEAS-2B) through activation of TRPV1 channels (vanilloid/capcaicin receptor) and subsequent calcium signaling [127]. TRPV1 was found to mediate effects of various PM, and the authors speculated that surface charge (zeta-potential) could predict the particles effects [128,129]. Later on, DEP was reported to regulate matrix metalloproteinase-1 (MMP-1) in bronchial epithelial cells, through activation of protease activated receptor-2 (PAR-2) and subsequent activation of calcium influx through TRPV4-channels in the plasma membrane [130]. This effect was attributed to the soluble organic fraction of DEP. Recent studies from our lab, suggest that PAR-2 signaling may be involved in DEP-induced regulation of IL-6, but not IL-8/CXCL8 (un-published results). However, it remains unclear whether PAR-2 is directly targeted by constituents of DEP or trans-activated as result of up-stream effects (Figure 3). TRPV1 and TRPV4 belong to the transient receptor potential (TRP) cation channels. As extensively reviewed elsewhere, several TRP-members (TRPA1, -V1, -V4 and -M8) have now emerged as potential “particle sensors”, and may play important roles in the adverse effects of combustion particles through regulation of calcium signaling [131]. Some of these receptors appear to be activated directly by combustion particles or soluble organics, such as TRPA1 and TRPV1, while others such as TRPV4 have been suggested to be activated more indirectly through transactivation processes (Figure 3) [131]. TRP-mediated calcium signaling, at least in the case of TRPV4, may seem to regulate gene transcription through activation of the ERK1/2 cascade [130]. In line with this notion we observed that the calcium-chelator BAPTA-AM blocks chemokine responses in BEAS-2B cells induced by a variety of compounds characteristic of ambient PM, including mineral particles, metals ions, PAHs, and endotoxin [123]. Effects on early calcium responses may therefore be an interesting endpoint to assess in the pursuit of initial triggering mechanisms of proinflammatory effects of ambient PM and combustion particles in particular. In this respect, it is also interesting to note that PAHs were recently found to activate β2-adrenergic receptors (β2AR) and calcium signaling, affecting regulation of IL-8/CXCL8 in BEAS-2B bronchial epithelial cells [132,133,134]. Moreover, cigarette smoke and cigarette smoke extracts were reported to induce transcription and secretion of MUC5A in bronchial epithelial cells (NCI-H292) through a pathway involving β2AR, β-arrestin2, and the MAPKs ERK1/2 and p38 [135]. Moreover, studies using β2-agonsists suggest that β2AR-signaling enhances IL-6 and CXCL8 responses in bronchial epithelial cells through a pathway involving cAMP and activation of C/EBP and/or CRE cites, but suppression of NF-κB activity [136,137]. Thus, β2AR may conceivably be another molecular target of organic constituents from combustion particles and ambient air PM (Figure 3). However, activation of β2AR alone does not appear to be sufficient to trigger transcription of proinflammatory cytokines and chemokines, including IL-6 and CXCL8 in bronchial epithelial cells, but rather acts in combination with other signaling mechanisms [134,136,137].

A reported discrepancy in gene expression patterns reported between coarse, fine, and ultrafine PM [138], suggests that these different size-fractions may induce effects through different mechanisms. Of notice, several studies have shown that coarse PM is a considerably more potent activator of proinflammatory responses than fine and ultrafine PM in lung tissue (REF). This has in many studies (at least partly) been attributed to a higher content of endotoxin in coarse PM, which mediates transcriptional activation of several proinflammatory genes through activation of TLR2/4 [139,140,141,142,143]. However, other studies have reported that the stronger proinflammatory effect of coarse PM was mediated through endotoxin-independent (but unidentified) mechanisms [144,145]. As noted previously, coarse PM may also contain mineral particles. These could possibly bind to cell surface receptors such as scavenger receptors and CD14. However, compared to the combustion-derived particles of fine and ultrafine PM, the mechanistic targets regulating the proinflammaory effects of coarse PM (PM_10–2.5_) are even less clear.

### 3.4. Intracellular Molecular Targets

Internalization also enables particles to interact directly with intracellular molecules. This may be particularly important for nano-sized particles such as engineered nanomaterials and ultrafine combustion particles. Single walled carbon nanotubes have been reported to interact with actin filaments causing actin bundling, associate with tubulin and chromatin and disrupt formation of the mitotic spindle, and act as ion channel blockers [146,147,148]. Moreover ultrafine, and to a lesser extent fine PM, have been found to localize in, and damage, mitochondria leading to increased formation of ROS [149]. However, it remains to be determined whether any of these effects may be of relevance for the regulation of proinflammatory genes in cells exposed to such particles.

It is, however, clear that soluble particle components such as organic chemicals and metals may affect proinflammatory responses through direct interaction with various intracellular targets. PAHs and dioxin-like compounds which may be adsorbed on urbane air particles are well known to activate the cytosolic aryl hydrocarbon receptor (AhR) which in its classical mode of action binds the AhR nuclear translocator (Arnt) and regulates expression of CYP1A1, -1A2 and -1B1 enzymes (Figure 3). AhR activation appears to be among the most sensitive targets of combustion particles and CYP1-expression is increased by very low concentrations of DEP [150,151], possibly already from a few nanograms per cm^2^ which is well below estimated deposition levels in the airways [35,150]. Increased CYP1-levels may contribute to increased formation of ROS and electrophilic metabolites from PAHs and quinone species (Figure 1). However, AhR also possesses several normal physiological functions and has amongst others been found to play a central role in regulation of inflammation and immune responses [152,153]. A study on human macrophage-like U937-cells, showed that COX-2 activation by urban dusts and DEP was mediated, at least in part, through AhR activation, while IL-6 and C-reactive protein (CRP) were regulated through AhR-independent mechanisms [154]. Moreover, the archetypical AhR ligand 2,3,7,8-tetrachlordibenzo-p-dioxin (TCDD) stimulated COX-2, IL-1β, MUC5AC, Clara cell secretory protein (CSSP) as well as other proinflammatory cytokines, both *in* *vitro* in Clara cell-like NCI-H441 cells and *in vivo* in mice [155]. Urban dust particles appeared to induce COX-2 and MUC5AC in NCH-H441 cells through a similar AhR-dependent mechanism [155]. However, in a study of different nitro-PAHs commonly found in DEP, we observed no correlation between the ability to stimulate CYP-responses and the ability to induced cytokine/chemokine gene-expression in bronchial epithelial BEAS-2B cells [73], and silencing of AhR rather enhanced 1-nitropyrene induced CXCL8 responses [156]. Similarly, Aung *et al.* [157] reported that the AhR-antagonist α-naphthoflavone suppressed CYP1A1 responses induced by urban air PM, but not COX-2 or CCL2 (MCP-1) in primary human endothelial cells. Resveratrol, which also act as an AhR-antagonist (but may also interfere with NOX-signaling and scavenge ROS), suppressed PM_10_-induced CYP1B1 and c-Jun responses, but not IL-6 or CXCL8 in A549 epithelial cells, suggesting that the latter were induced through AhR-independent mechanisms [158]. Furthermore, in DEP-exposed BEAS-2B cells we observed an inverse relation between CYP1A1 and IL-6/CXCL8 responses. CYP1A1 was induced at extremely low concentrations of DEP, but reduced at higher concentrations when IL-6 and CXCL8 increased [150]. Moreover, while the proinflammatory components of DEP could be extracted by methanol washing, resulting in reduced effects of the residual DEP on IL-6 and CXCL8, these washed particles displayed enhanced ability to stimulate CYP1A1 [33]. Recent findings from our laboratory show that interference with AhR signaling by siRNA mediated transient knock-down or by use of pharmacological inhibitors (CH223191 and 6,2,4-trimethoxyflavone) suppressed DEP-induced CYP1A1 expression but not IL-6 or CXCL8 responses in BEAS-2B cells (unpublished results). Of note, there is an extensive cross-talk between AhR and the proinflammatory transcription factor NF-kB, and the two pathways appear mutually suppressive [159]. AhR may also be directly involved in transcriptional regulation of several proinflammatory genes, either through binding of AhR:Arnt to classical XRE-sites or through binding of AhR:NF-κB-dimers to κB-sites or other sequences in the promotor regions [160,161,162,163,164,165]. However, the role of AhR in regulation of inflammation appears highly complex and cell-dependent [156,160,161,162,163,165,166,167,168]. In fact, AhR ligands may act both pro- and anti-inflammatory even within the same cell line (BEAS-2B) dependent on combinatory co-stimuli, and constitutive AhR-activity seems to affect phosphorylation of p65 at serine 536, which promotes coupling of p65 to the basal transcriptional machinery [156,169]. AhR also appear to suppress other yet unidentified signaling pathways involved in regulation of CXCL8 and CCL5 (RANTES) in BEAS-2B cells [156], and has been reported to suppress both AP-1 and C/EBP in other cell types [170,171]. In addition to the effects on regulation of proinflammatory genes, ligand-activation of AhR also appears to be an important mechanism in maturation and activation of immune cells [170,172,173], which conceivably could be affected by exposure to DEP and other combustion particles. A recent study suggest that DEP extracts (NIST SRM 1975) and B[*a*]P may affect Th17/Th22 polarization by suppressing IL-17 and stimulating IL-22 in peripheral blood mononuclear cells (PBMCs) from normal and asthmatic subjects [174]. DEP is also known to promote Th2-responses, but the molecular mechanisms for this effect remain to be clarified [175,176]. In addition to this, Ferecatu *et al.* [177] showed that PM_2.5_ could suppress apoptosis through an AhR-dependent mechanism, and speculated that anti-apoptotic effects of PM_2.5_ could prolong inflammatory effects induced by air pollution exposure. Thus, AhR is clearly a highly sensitive target of PAHs and/or dioxin-like constituents of DEP and other combustion-derived particulates, and likely to be of considerable importance in immunotoxicity. However, due to the complexity of AhR-signaling, its role in the transcriptional regulation of proinflammatory genes induced by particle-exposure is still not completely understood.

Several metals such as zinc (Zn), arsenic (As), nickel (Ni), iron (Fe), copper (Cu), vanadium (V) manganese (Mn), lead (Pb), and cadmium (Cd) have been associated with health effects induced by PM [178,179]. Various particle-associated metals have been reported to activate MAPKs and NF-κB signaling in bronchial epithelial cells and other cell types, and may thereby affect the expression of cytokines and chemokines as well as other proinflammatory genes [180,181,182,183]. Generation of oxidative stress has been considered as one of the major mechanisms behind heavy metal toxicity, as already discussed, and heavy metals have been shown to disturb signal pathways and modulate transcription factors important in regulation of cytokines [184,185]. Furthermore, toxicity of heavy metals may be induced either by their direct binding with cysteinyl thiol-groups of proteins/enzymes, thereby causing perturbations in their three-dimensional conformations, inactivation of catalytic sites or by displacement of essential metal ions. In both situations the protein/ enzyme functions are greatly compromised, which might result in disturbed cellular functions. The relative ability to augment the phosphorylation or inhibiting dephosphorylation represents an important mechanism through which many metal ions may activate intracellular signaling pathways and affect regulation of gene expression. In particular, protein tyrosine phosphatases (PTPs) appear to be common targets of several metals. PTPs act as negative regulators of signaling by dephosphorylating and inactivating kinases. Both V and Zn are well-known PTP inhibitors, and PTPs are reported targets of V and Zn in human airway epithelial cells [186,187]. In fact, it has been proposed that intracellular Zn^2+^ functions as an endogenous second messenger in PTP inhibition [188,189], analogous to the role of ROS described later. Inhibition of phosphatase activity has been suggested as an initiating event in MAPK activation induced by Zn in BEAS-2B cells [182]. Zn is also reported to inhibit the EGFR-specific PTP [190]. In addition to inhibit dephosphorylation, metals might also augment phosphorylation in signaling pathways, and Zn is reported to induce EGFR transactivation by c-Src in a human epidermoid cell line (A431) [191]. A thorough review on the mechanisms regulating pulmonary toxicity of Zn is available from Wu *et al.* [192], for those interested in further details on this subject. Of notice, a majority of these studies concern the role of Zn and V as mediators of effects from ambient air PM or welding fumes. However, soluble nanoparticles of Zn- and V-oxides releasing metal ions would be likely to affect regulation of proinflammatory genes through similar mechanisms.

## 4. Central Signaling Pathways and Processes Involved in Particle-Induced Activation of Proinflammatory Genes

### 4.1. Role of Endogenous ROS Formation in Cellular Signaling and Inflammation

As previously described the “oxidative stress paradigm” has been a dominating theory for how particles trigger proinflammatory genes and other cellular responses. However, the traditional oxidative stress paradigm has been under intense debate within free radical biology, and is suggested to be replaced by the concept of “redox biology” which incorporates the view that redox-active mediators including ROS and RNS act as site-specific mediators of cell-signaling and central regulators of inflammatory responses [193,194]. This emerging perspective on redox-regulation as a normal physiological process suggests that many of the observed effects of antioxidant treatment on particle-induced inflammatory responses, may possibly be due to interference with normal redox-signaling rather than interference with what traditionally has been considered “oxidative stress responses”. In the present chapter the role of secondary, endogenous ROS production is discussed in relation to its role in the signal transduction pathways leading to transcriptional activation of proinflammatory genes in particle-exposed cells and tissues.

**Figure 4:**
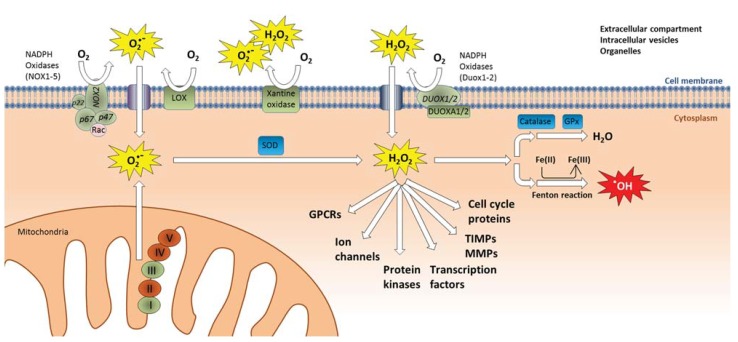
Intracellular sources of endogenous ROS formation. Membrane-bound oxidases such as NOX1-5 and LOX as well as the mitochondrial electron transport chain (Complex I and III) produce superoxide (O_2_^●−^), xanthine oxidases may produce both superoxide and hydrogen peroxide (H_2_O_2_), while DUOX1/2 produce hydrogen peroxide. Oxidases induce ROS formation on the outside of cellular membranes, in the extracellular compartment, or inside intracellular vesicles and organelles such as endosomes, lysosomes, and endoplasmic reticulum. Superoxide and hydrogen peroxide may be transported through the membranes into the cytoplasm through anion channels and aquaporins, respectively. Superoxide is converted by SOD into hydrogen peroxide, which functions as a central intracellular second messenger in a variety of signaling pathways. Hydrogen peroxide is then rapidly inactivated and converted to H_2_O by catalase or GPx. Excess levels of redox active transition metals such as Fe(II) may convert hydrogen peroxide into highly reactive hydroxyl radicals (^●^OH) through the Fenton-reaction, leading to oxidative damage on membrane lipids, proteins and DNA. Abbreviations: DUOX, dual oxidases; DUOXA, dual oxidase A; GPCRs, G-protein coupled receptors; GPx, glutathione peroxidase; LOX, lipoxygenases; NOX, NADPH oxidases; MMPs, matrix metalloproteinases; SOD, superoxide dismutase; TIMPs, tissue inhibitors of metalloproteinases.

**Figure 5:**
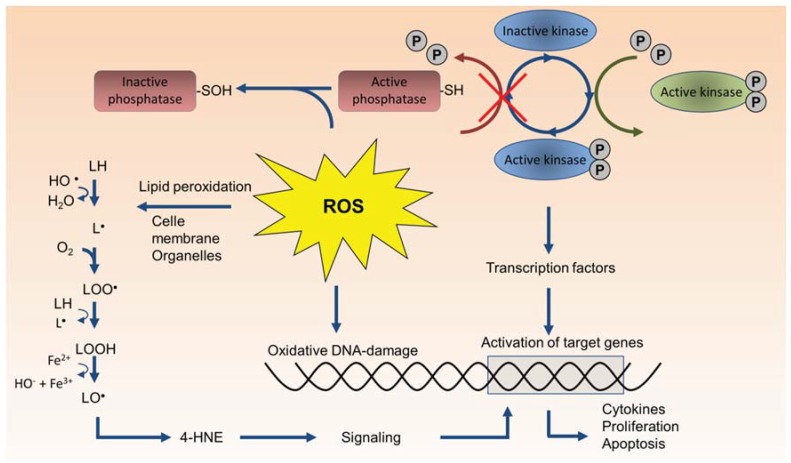
Effects of ROS on cellular components. Kinases are a large group of intracellular signaling proteins that are activated by phosphorylation from up-stream kinases and elicit their effects by phosphorylation of down-stream, targets. Kinase activity is suppressed by protein phosphatases. ROS (H_2_O_2_) oxidizes SH-groups on phosphatases leading to reversible inactivation of the phosphatase. This allows increased kinase activation and increased signaling. At higher concentrations H_2_O_2_ may further oxidize thiolate anions into sulfinic (SO_2_H) or sulfonic (SO_3_H) species leading to the irreversible protein damage. Excessive oxidative stress (in particular from ^●^OH) may cause oxidative damage to proteins, DNA and membrane lipids (lipid peroxidation). Lipid peroxidation may result in damage to cell membranes and organelles, and formation of reactive oxygenated α,β-unsaturated aldehydes such as 4-hydroxy nonenal (4-HNE).

ROS (and also RNS) act as central endogenous second-messengers and are involved in regulation of complex cellular processes such as mitogenic signal transduction, phagocytosis, gene expression, regulation of cell proliferation, replicative senescence, and apoptosis, and are essential effectors in the inflammatory response [195,196,197,198,199,200]. Some effects have been attributed to reversible oxidation of critical thiol-groups within signaling proteins by hydrogen peroxide (H_2_O_2_). Cells can produce H_2_O_2_ directly or indirectly by producing superoxide, O_2_^●−^, which is rapidly converted to H_2_O_2_ by superoxide dismutases (SOD), as shown in Figure 4 [196,201,202,203,204]. Reversible oxidation of cysteine residues by nanomolar concentrations of H_2_O_2_ is considered a main mechanism of redox signaling [199]. Oxidation of cysteine residues inactivates PTPs which is essential in order to allow phosphorylation and activation of protein kinases (Figure 3) [196,197]. Activation of a variety of RTKs therefore involves production of ROS [196], and even G-protein coupled receptors may be under redox-regulation, despite their lack of kinase activity [205]. The primary source of ROS in receptor-mediated signaling appears to be plasma membrane oxidases, mainly NOX, which are rapidly activated and inactivated [196], but also mitochondrial ROS formation appears to play a central role in regulation of proinflammatory cytokines [203]. More recent studies further suggest that even electrophilic lipid-species such as aldehydes and α-β-unsaturated carbonyls may react with cysteines in a reversible manner and serve specific signaling functions in inflammatory conditions [206,207]. The specificity of this system is enabled by highly compartmentalized production and inactivation of reactive species. Endogenous antioxidants and related enzymes may therefore play a primary role in controlling cell signaling by insulating cysteine residues and other cell signaling domains from uncontrolled activation by oxidants or electrophiles [206,207]. Notably, higher concentrations of oxidants and electrophiles may lead to some of the irreversible damage observed during excessive oxidative stress. H_2_O_2_ may also be transformed into highly reactive hydroxyl radicals (^●^OH) through Fenton-reactions, leading to excessive oxidative stress, lipid peroxidation, and oxidative DNA-damage (Figure 4 and Figure 5). However, the relevance of such oxidative stress responses in the pathology of inflammatory diseases has been questioned [194].

Many proinflammatory genes often assessed in particle toxicology such as CXCL8 (IL-8), IL-6, and COX-2, are regulated through autocrine and paracrine signaling loops initiated by the release of early cytokines such as IL-1α/β and TNF-α [208,209,210,211,212]. IL-1 and TNF-α are produced as immature pro-forms that are cleaved prior to the release of their active mature forms. Notably, both transcriptional activation and cleavage/release of IL-1 and TNF-α as well as signaling from IL-1 and TNF receptors (IL1R and TNFR) involve multiple redox-regulated steps [213,214,215,216,217,218,219,220,221,222,223,224,225,226]. Similarly, EGFR signaling which appears to be important in regulation of the magnitude of proinflammatory responses induced by various particles and air pollution components represents another key pathway that is under considerable redox regulation [227,228,229,230]. Thus, it seems that any particle-induced effect leading to transcriptional activation of proinflammatory cytokines and chemokines such as CXCL8 and IL-6 in airway epithelial cells would be expected to be inhibited by antioxidant treatment irrespective of the particles’ own oxidative capacity, since ROS appear to be involved as central second messengers acting at multiple levels in a multitude of the signaling pathways that regulate these proinflammatory genes. Consistent with this, Barret *et al.* [210] reported that crystalline silica induced MIP-2 release from alveolar type-II cells (MLE-15 cells) were mediated by TNF-α-induced oxidant stress. Silica exposure induced TNF-α release through a non-oxidant mechanism, but TNF-α subsequently stimulated ROS formation in an autocrine manner, leading to increased production and release of MIP-2 [210]. Driscoll *et al.* [231] later reported that silica-induced MIP-2 responses were mediated by mitochondrial-derived ROS generation in type-II epithelial RLE-6TN cells. Moreover, by use of pharmacological inhibitors and knock-out mice, both NOX and inducible nitric oxide synthase (iNOS) were found to be involved in cytokine responses induced by different mineral particles and suspended particulate matter [232]. Also soluble metal ions may indirectly lead to generation of ROS. This may partly be due to depletion of the cell’s antioxidants, particular thiol-containing antioxidants (e.g., GSH) and enzymes involved in antioxidant mechanisms [184,185]. Through ion-displacement, redox-inactive metals may also release redox-active metal ions (Fe and Cu) from cellular proteins, and thereby contribute to oxidative stress through Fenton- and Haber-Weiss-type reactions [178]. In addition, metal ions may cause mitochondrial dysfunction by inhibition of electron transfer chain or through reactions with redox active enzymes such as NADPH oxidases [184,185]. Indeed, a range of *in vitro* and *in vivo* studies using pharmacological inhibitors, RNA interference, and genetic knock-out models, suggests that increases in intracellular ROS levels in exposed cells as well as tissue levels of ROS in the lung and vasculature of particle-exposed animals, predominately arise from endogenous redox-processes, such as activation of oxidases or mitochondrial respiration [231,232,233,234,235,236,237,238,239,240,241,242]. Although the majority of these studies did not address mechanisms of particle-induced inflammation, it seems likely that this endogenous ROS generation may also affect regulation of proinflammatory genes. Furthermore, a study utilizing intracellular and extracellular antioxidants was able to show that activation of Akt/ERK signaling in rat lung epithelial cells (RLE-6TN cells) by ultrafine carbon black and ferric sulfate was mainly due to intracellular ROS generation rather than extracellular (abiotic) ROS formation [59].

In addition to its role in signal transduction, ROS is produced during a number of different cellular processes including cell death, phagocytosis, and metabolism. Therefore, in a particle-exposed cell, increases in ROS levels may potentially originate from a number of different sources including direct ROS formation by the particle surface, metabolic activation, and redox cycling of particle-bound organics such as PAHs and quinones, formation of more potent ROS by Fenton-reactive transition metals, as well as activation of cellular oxidases and mitochondrial respiration (Figure 1). With all these possible sources of ROS inside and outside of a particle-exposed cell, delineating the precise effect of an antioxidant is complicated. In the *in vivo* situation, the picture is further complicated by the central role of oxidative burst in activated immune cells, and the fact that inflammatory reactions results in a certain level of oxidative stress. However, the collective evidence appear to suggest that the main contribution of ROS and other reactive species in the onset and regulation of proinflammatory responses and toxicity by particles, may arise from changes in the cell’s endogenous redox processes.

### 4.2. Inflammasome Activation—Linking Lysosomal Rupture to Proinflammatory Responses

**Figure 6:**
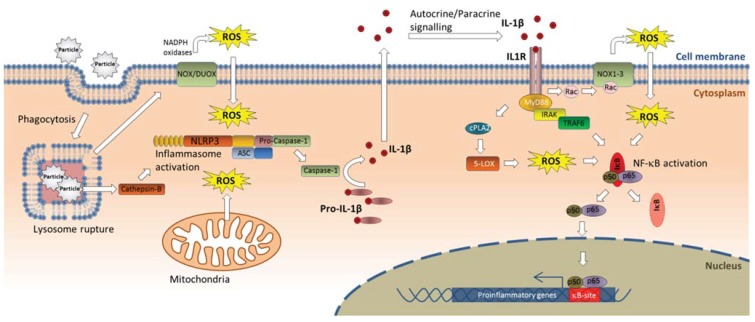
Particle-induced IL-1β release through activation of the NLPR3 inflammasome. Phagocytosed particles end up in lysosomes, where they may cause damage to the lysosomal membrane. This leads to activation of the NLRP3 inflammasome through a mechanism that may involve cathepsin-B release and ROS-generation by membrane-bound NOX and/or mitochondria. NLRP3 associates with ASC and activates caspase-1 which cleaves pro-IL-1β into the mature IL-1β. Released IL-1β may act in autocrine and/or paracrine manner, bind the IL-1R and stimulate production of a variety of proinflammatory genes, amongst others through activation of NF-κB signaling. Abbreviations: ASC, apoptosis-associated speck-like protein containing a carboxy-terminal CARD; cPLA2, cytosolic phospholipase A2; DUOX, dual oxidases; IL-1β, interleukin-1β; IL-1R, IL-1 receptor; IκB, inhibitor of κB; IRAK, interleukin-1 receptor-associated kinase;5-LOX, 5-lipoxygenase; MyD88, myeloid differentiation primary response gene 88; NF-κB, nuclear factor-κB; NLRP3, NACHT, LRR and PYD domains-containing protein 3; NOX, NADPH oxidases; ROS, reactive oxygen species; TRAF6, TNF receptor associated factor 6.

One of the biggest breakthroughs in our understanding of inflammation by crystalline particles came from outside the field of particle toxicology. In what has rightfully been termed “a scientific *tour de force*” [243] the late Jürg Tschopp and co-workers, showed that the central mechanism driving gouty arthritis was mediated by MSU crystals activating the NACHT, LRR, and PYD domains-containing protein 3 (NLRP3 or NALP3), leading to assembly of the inflammasome. The NLRP3 inflammasome belongs to a family of intracellular protein complexes that recognizes so-called danger associated molecular patterns (DAMPs) and regulates activation of caspase-1. Caspase-1 cleaves pro-IL-1β and pro-IL-18 leading to secretion of IL-1β and IL-18 in their active forms, subsequently eliciting a potent inflammatory response [244,245,246,247,248,249]. MSU crystals and other endogenously formed crystals (calcium pyrophosphate dehydrate crystals responsible for pseudogout and cholesterol crystals in atherosclerotic plaques) induced IL-1β and IL-18 from THP-1 cells by mediating NLRP3-dependent caspase-1 activation, and knock-out of various components of the NLRP3/caspase-1 cascade impaired the induction of IL-1β activation and neutrophil influx [245,250,251]. Moreover, it was quickly realized that the NLRP3/Caspase-1/IL-1β-cascade was also responsible for the proinflammatory effects of crystalline silica, asbestos, and aluminum salts in macrophages and other phagocytic immune cells [252,253], and NLRP3 was reported to be essential for development of silicosis in mice [252,254]. An overview of the mechanisms involved in NLRP activation and IL-1β signaling is given in Figure 6.

The mechanism of crystal-induced NLRP3 activation appears to be linked to destabilization and rupture of lysosomes leading to release of cathepsin B, amyloid-β, and activation of NADPH oxidases as well as ROS generation, and also appears to involve K^+^ efflux, Ca^2+^ influx [251,252,253,255,256]. Interestingly, MSU crystals which are formed after release of uric acid from dying or injured cells, have been proposed to represent an endogenous danger signal in immunity and inflammation [257].Thus, the considerable proinflammatory activity of crystalline silica and asbestos may partly be due to their crystal structure mimicking the effects of endogenous danger signals. Later it became clear that a number of other natural and man-made particulates are also “sensed” by the NLRP3 inflammasome, including nanoparticles of carbon, silver, quartz, and polystyrene as well as single- and double-walled carbon nanotubes [258,259,260,261,262,263]. Thus, delineating the role of NLRP3 has provided an explanation for how lysosomal rupture triggers inflammatory reactions and cytotoxicity in phagocytes and has provided treatment strategies for crystalline particle-induced diseases such as gout and possibly also for silicosis and other particle-induced effects. Of note, airway epithelial cells also appear to engulf particulates such as crystalline silica [95]. Recent studies suggest that both crystalline silica and carbon nanotubes activate the NLRP3 inflammasome in airway epithelial cells leading to cleavage and release of mature IL-1β showing that this mechanism is not restricted to classical phagocytic cells [264,265].

### 4.3. The EGF Receptor—A Regulator of the Magnitude of Particle-Induced Inflammation?

The EGFR signaling cascade represents another key intermediate pathway involved in regulation of proinflammatory genes. Membrane bound pro-forms of EGFR ligands such as TGF-α, amphiregulin, and HB-EGF are cleaved and released by the metallo-protease TNF-α converting enzyme (TACE or ADAM17), leading to EGFR-ligand release and activation of EGFR [227,228,266]. Studies, amongst others from our laboratory, show that CXCL8 (and likely also IL-6 responses) in airway epithelial cells induced by quartz, nano-silica, diesel exhaust particles, and ambient air PM, as well as a number of air pollution components including PAHs, metals and endotoxin, are dependent on signaling through the TACE/TGFα/EGFR-cascade [95,120,122,123,124,125,267]. These findings suggest that TACE and EGFR may be central regulators of the magnitude of cytokine/chemokine responses in airway epithelial cells. TACE/EGFR signaling also regulates mucin production in epithelial cells, which contributes to exacerbation of asthma, COPD, and cystic fibrosis [228,268]. Moreover, TACE also cleaves and activates proteins involved in adhesion and transmigration of leukocytes across the endothelium, including various adhesion and tight-junction molecules [269]. Thus, the TACE/EGFR-cascade regulates a number of processes that participate in the promotion of inflammation in the airways. Of particular interest, EGFR-expression appears to be increased in the airway epithelium of asthmatics, smokers and patients with COPD and CF, and the pulmonary expression of EGFR and CXCL8 correlates in patients with severe asthma [270]. Similarly, it has been reported from animal-models that tissue-levels of TACE are elevated in COPD [271], and that TACE-inhibitors decrease neutrophil influx in both allergic and non-allergic airway inflammation [220]. It is therefore tempting to speculate that such enhanced TACE- and EGFR-expression may represent susceptibility factors for development or exacerbation of inflammatory disease by inhaled particulates and other air pollutants.

### 4.4. Proinflammatory Transcription Factors Activated by Particle Exposure

Transcriptional activation of proinflammatory genes is elicited through combined activation of different transcription factors. The classical NF-κB, manifested by the p50:p65 (or p50:RelA) hetero-dimer, has received most attention both in particle toxicology as well as in innate immunity in general, as illustrated by several reviews [35,198,272,273]. NF-κB is generally viewed as a ROS-dependent pathway involved in the induction of inflammatory cytokines such as IL-1-family proteins, TNF-α, IL-6, and CXCL8/IL-8 [35]. However, the role of ROS in NF-κB signaling appear to be highly cell specific, and it is not clear whether oxidant directly triggers the components of the NF-κB pathway or rather affects up-stream targets [274]. Several studies show that ROS can actually inhibit NF-κB signaling in airway epithelial cells through interactions with cysteine residues of IKKβ [274]. The ability of particles to induce inflammatory cytokines has often been linked to the combined action of NF-κB and MAPKs. In fact, activation of MAPKs and NF-κB has been observed as a response to all the main groups of respirable particles, among them cigarette smoke (CS), silica (SiO2), asbestos, carbon nanoparticles, DEP, and ambient PM. MAPKs such as ERK and JNK may partly mediate their effect through activation of AP-1-transcription factors, such as c-jun and c-fos proteins. Of interest, Tal *et al.* [275] found that DEP with a different content of organic constituents induced CXCL8/IL-8 responses through different mechanisms. DEP with a high organic content induced IL-8 through AP-1 activation, whereas low organic DEP induced IL-8 expression through a mechanisms that was predominately NF-κB-dependent. MAPKs also regulate the nuclear factor ATF2. In response to exposure of mice to residual oil fly ash (ROFA) the activation of ATF2 was observed in the bronchial and alveolar epithelium in addition to alveolar macrophages, whereas NF-κB activation was only found in the bronchial epithelium [276]. The STAT proteins in the lung seem to be mainly associated with the regulation of adaptive immunity response [277]. DEP have been found to trigger STAT-3 activation through EGFR and Src [278]. DEP induced eotaxin was found to be STAT-6 independent and instead to require NF-κB activation [279]. The early growth response gene-1 (Egr-1) protein seems to be involved in the up-regulation of proinflammatory cytokines after cigarette smoke (CS) exposure of the lung, through a pathway involving up-regulation of the receptor for advanced glycation end-products (RAGE) [280,281]. Similar mechanisms may also be involved in effects from DEP-exposure [282]. Moreover, the combined exposure to ultrafine particles and cigarette smoke extract leads to an activation of MAPK, Egr-1 and production of IL-6 in mouse endothelial cells [283]. However, researches in the field have mainly focused their attention on NF-κB and AP-1. Knowledge on the mechanisms involved in transcriptional activation of proinflammatory genes by particles and particle components is therefore still relatively scarce.

### 4.5. Cytotoxicity as Triggering Mechanism for Proinflammatory Responses

The majority of this review has focused on the initial molecular targets of particles and particle components that are directly linked to transcriptional regulation of proinflammatory genes through intracellular signaling pathways. However, particles may also trigger activation of proinflammatory responses through more indirect responses. When cells are injured or die, they release danger signals to alert the host and may trigger inflammation. In particular, necrosis is a well-known cause of inflammation, but also apoptosis which normally is considered anti-inflammatory, may under certain circumstances activate inflammatory stimuli. Various DAMPs (damage associated molecular patterns) are released from necrotic cells including eATP, oxLDL, oxDNA, ssRNA, dsRNA, cholesterol, and MSU crystals, as well as amyloid-β, HMGB1, HSP, and can bind to various receptors (TLR and RAGE) on macrophage and trigger cytokine and chemokine formation. Such mediators may in this way aggravate injury by recruiting cytotoxic neutrophils to the tissue. The role of cell death in inflammation is beyond the scope of this manuscript and has been extensively reviewed elsewhere [284]. The important aspect is that any particle that induces necrosis and in some cases also apoptosis, or in other ways damages cellular membranes leading to leakage of cytosolic mediators, may also be anticipated to trigger inflammatory reactions through the same mechanisms. This is underscored by a study using bleomycin, which showed that release of uric acid (the precursor of MSU crystals) from injured cells activated the NLRP3 inflammasome, and caused IL-1β production, lung inflammation, and fibrosis [285].

To what extent quartz, MSU crystals and other particles can damage lysosomal membranes and trigger activation of NLRP3/Caspase-1/IL-1β cascade, independent of cytotoxicity is not very clear from the existing literature. However, a recent study suggests that release of endogenous IL-1α represents an even more early and crucial event that determines lung inflammatory responses to particles in mice [286]. Most interestingly, it was reported that IL-1α constitutively expressed in resident alveolar macrophages was released from necrotic macrophages and could serve as an alarmin after silica exposure. IL-1α was found to be a potent activating stimulus required for surrounding macrophages to express the biologically inactive precursor IL-1β (pro-IL-1β), which next was cleaved and secreted as mature and bioactive IL-1β by silica-induced activation of inflammasome [286]. If this is the case, inflammasome activation and IL-1β responses in particle-exposed phagocytes may be closely connected with cytotoxicity and cell death. Such a notion would be in line with the age-old perception that the continuous killing of macrophage by lysosomal rupture is essential in pulmonary quartz toxicity [84,87,88].

## 5. Conclusions

Clarifying the initial mechanisms through which particles trigger responses that ultimately regulate proinflammatory genes is at the core of particle toxicology. This may in the long term lead to more efficient abatement strategies to reduce emissions of the most hazardous air pollutants, to reduce toxicity of engineered nanomaterials, and perhaps also to identify therapeutic targets that can be used in preventive treatment of susceptible groups.

Particle toxicology covers the effects of a diverse range of compounds, which broadly can be divided into the following categories: solid particles and fibers, soluble particles, and complex particles which act as carriers of an abundance of organic and inorganic compounds. Reported molecular triggering mechanisms involving activation of proinflammatory genes and the onset of inflammatory reactions by particles or soluble particle components can further be categorized into direct formation of ROS with subsequent oxidative stress, interaction with the lipid layer of cellular membranes, activation of cell surface receptors, and direct interactions with intracellular molecular targets. An overview of the oxidant mediated triggering mechanisms for particle-induced effects are given in Figure 1, Figure 4 and Figure 5, while the non-oxidant mechanisms are illustrated in Figure 2 and Figure 3. Importantly, ROS act as a central second-messenger in a variety of signaling pathways. Thus, even non-oxidant mediated triggering mechanisms are likely to activate downstream redox-regulated events.

A number of intriguing discoveries have broadened our understanding of the molecular mechanisms underlying particles-induced inflammation in the airways. The most groundbreaking revelation is possibly the central role played by the NLRP3-inflammasome/caspase-1/IL-1β-cascade as a potential master switch, regulating cytokine and chemokine responses by crystalline particles and other solid particulates. This has provided a mechanistic link between lysosomal rupture in phagocytes and the onset of inflammation in the airways. However, the recent reporting that IL-1α release from dying or injured macrophages precedes IL-1β activation [286], suggests that the complete mechanisms regulating particle-induced NLRP3 activation and IL-1β release still remains to be clarified. Furthermore, redox-regulated processes clearly play a central part in mediating the proinflammatory effects of particles. Any particle that induces a sufficient level of ROS outside or inside a cell will, without doubt, induce a cellular response. The question is how much of the particle-induced effects are due to ROS formation and to what extent the ROS involved in particle-induced effects arise directly from the particles and particle components or indirectly from activation of cellular ROS generation. In recent years, a variety of more specific molecular triggering mechanisms for effects of particles and particle components has been described in the literature. However, for several of the receptors reported to be activated by particle exposure, it still remains to determine whether these are direct targets of particles or particle components or whether the nature of their activation is more indirect and rather results from transactivation processes, for instance through ROS-formation or more unspecific effects on membrane lipids affecting raft assembly. Furthermore, to this date, few if any studies have compared the relative importance of different triggering mechanisms (ROS, receptors, membrane fluidity) across a broader panel of proinflammatory genes.

Considering the vast numbers of materials and chemicals that are encompassed by the field of particle toxicology, it can reasonably be assumed that many aspects of the molecular mechanisms involved in particle-induced inflammation remain to be clarified. Moreover, to fully characterize the role of proinflammatory genes and inflammation in the onset and development of the distinctly different pathologies induced by different particles ranging from fibrotic diseases by mineral particles and fibers to cardiovascular effects from ambient PM will be a considerable task. Researchers in the field should therefore have their hands full for yet another decade.
